# The Need for Regulatory Certainty on Medical AI: Lessons From the OpenEvidence Geoblocking in the EU and UK Over the EU AI Act

**DOI:** 10.1016/j.lanepe.2026.101718

**Published:** 2026-05-23

**Authors:** Luigi De Angelis, Francesca Aurora Sacchi, Ciro Pappalardo, Giacomo Diedenhofen, Nicola Cosentino, Francesco Baglivo

**Affiliations:** a Italian Society of Artificial Intelligence in Medicine (SIIAM, Società Italiana Intelligenza Artificiale in Medicina), Rome, Italy; b Department of Translational Research and New Technologies in Medicine and Surgery, University of Pisa, Pisa, Italy; c Epidemiological Surveillance Unit, Department of Prevention, South Tyrol Health Authority, Bolzano, Italy; d Department of Perioperative Cardiology and Cardiovascular Imaging, Centro Cardiologico Monzino IRCCS, Milan, Italy

As of early 2026, retrieval-augmented generation (RAG) has become the standard architecture for medical applications of generative artificial intelligence. By grounding language models' output in a curated corpus, RAG mitigates the hallucinations that had previously restricted its clinical use and now underpins the principal evidence synthesis platforms publicly available.^1^

On the 27th April 2026, OpenEvidence, a platform reporting some 18 million clinical consultations a month, suddenly became unavailable across the European Union and the United Kingdom. The page that replaced the service attributed the withdrawal to “regulatory uncertainty” related to the EU Artificial Intelligence Act, referencing a 2023 open letter signed by around 150 European executives on AI regulation. It also initially provided contact details for specific Members of the European Parliament, which were subsequently removed (as of April 29, 2026). The episode elicits a comparison with the March 2023 suspension of ChatGPT in Italy by the national Privacy Authority,^2^ though the two diverge in their initiating actor: a public authority in the earlier case, the service provider itself in the present one.

Conceived largely before generative AI became a commodity, the AI Act faces the challenge of applying rules to a landscape that rapidly shifts. Critics argue that while the AI Act’s risk-based framework is conceptually sound, its current implementation lacks the dynamic risk-benefit analysis required, often defaulting to a bureaucratic approach that may be as ineffective as it is restrictive.^3^ This scenario is further complicated by the AI Act's interaction with the Medical Device Regulation, as many AI-based clinical tools fall under both frameworks, creating regulatory overlap and grey areas.^4^

To bridge this gap the EU proposed a staggered implementation of the AI Act, with most requirements becoming applicable by August 2026 and some obligations extended to August 2027. Moreover, the EU introduced the AI Pact: a voluntary initiative for proactive safeguard implementation.^5^ Notably, the Pact signals a pragmatic shift in stance: former critics such as Capgemini and Orange, original signatories of the 2023 letter, have now joined alongside global leaders like Google, Microsoft, and OpenAI in collaborative development. In contrast, OpenEvidence remained absent from both the critical open letter and the collaborative AI Pact, choosing instead a voluntary preventive shutdown. By disabling access for clinicians and directing them toward political decision-makers, the provider appears to be leveraging professional frustration to influence the legislative process, effectively encouraging a form of “bottom-up” lobbying.

This episode highlights a growing need for solutions that are designed and operated within the EU, ensuring data is processed locally and regulation compliance is embedded by design. At the same time, regulatory uncertainty and grey areas could hinder innovation, as ambiguity, more than regulation itself, is the real constraint. A strategic commitment is needed to harmonize the existing legislative frameworks, aiming to a single, enforceable lex specialis for medical AI. This unified standard would provide essential certainty for innovators, while simultaneously strengthening auditability and user protection through robust transparency and accountability. While current regulations are not without flaws, an “innovation at all costs” approach is not acceptable in the clinical context. A shared strategic roadmap is needed, one that balances technological advancement with the paramount importance of patient safety.

## Contributors

LDA and FB conceptualized the article. All authors reviewed the literature, wrote the first draft, reviewed, and provided edits. All authors validated the final version of the article.

## Declaration of generative AI and AI-assisted technologies in the manuscript preparation process

During the preparation of this work the authors used Grammarly, ChatGPT, Gemini and Claude in order to perform English grammar checks. After using this tool/service, the authors reviewed and edited the content as needed and take full responsibility for the content of the published article.

## Declaration of interests

LDA, FB, CP, GD, and FAS are board members of the Italian Society of Artificial Intelligence in Medicine (SIIAM), a not for profit scientific society. FAS is contracted by Sanofi. LDA is a Board Member and Chief Research Officer of HumanTruths, Inc. NC has a fiduciary role in Società Italiana di Cardiologia (no-profit) and received consulting fees from Alnalym, AstraZeneca, Aurora Biopharma, Bayer, Boehringer Ingelheim, Eli Lilly, and Pfizer. CP is a medical consultant on digital health projects at HSPI Spa.
